# Multiple Sclerosis Therapy With Disease-Modifying Treatments in Germany: The PEARL (ProspEctive phArmacoeconomic cohoRt evaluation) Noninterventional Study Protocol

**DOI:** 10.2196/resprot.4473

**Published:** 2016-02-04

**Authors:** Stefan Viktor Vormfelde, Sonja Ortler, Tjalf Ziemssen

**Affiliations:** ^1^ Novartis Pharma GmbH Clinical Research in Neuroscience Nuremberg Germany; ^2^ Klinik und Poliklinik für Neurologie Zentrum für Klinische Neurowissenschaften Universitätsklinikum Carl Gustav Carus, Technische Universität Dresden Dresden Germany

**Keywords:** multiple sclerosis, outpatient care, efficacy, safety, pharmacoeconomics, disease-modifying therapies

## Abstract

**Background:**

Patients with multiple sclerosis (MS) require long-term therapy and have a wide variety of needs for health-related support. The efficacy and safety of MS therapy, as assessed by both clinicians and patients, are important parameters that need to be considered. However, few studies combine data on efficacy and safety outcomes with pharmacoeconomic data.

**Objective:**

Here, we present the study design of the ProspEctive phArmacoeconomic cohoRt evaluation (PEARL), a prospective, multicenter, noninterventional cohort study on patients with relapsing-remitting MS (RRMS) treated with disease-modifying treatments (DMTs).

**Methods:**

During a prospective observational phase of 24 months per patient, PEARL evaluated clinical and patient-perceived efficacy and safety measures, as well as pharmacoeconomic data on RRMS patients treated with DMTs—interferon beta and glatiramer acetate. Measurements of the patients' perceptions included the assessment of patient-reported quality of life, treatment satisfaction, and compliance. The study was planned to include 1800 outpatients from 180 German neurological practices who had continuously been treated with an approved DMT for at least 30 days. The primary statistical analyses of the PEARL study will be descriptive. Particular focus will be on specific subgroups, such as patients who switched DMTs during therapy and patients with disease worsening or disease activity. Subgroups will be compared using stratified analyses.

**Results:**

Data collection for PEARL started in September 2010 and ended in July 2013. As of July 2015, the study is completed and is currently being analyzed and written up.

**Conclusions:**

PEARL is evaluating both the health status and resource utilization of RRMS patients treated with DMTs in Germany. The combination of pharmacoeconomic data with clinical and patients' self-perceived efficacy and safety outcomes will add useful information to the currently incomplete picture of the overall RRMS burden in Germany.

## Introduction

Multiple sclerosis (MS) is an inflammatory, demyelinating disease of the central nervous system with considerable physical, treatment-related, and economic consequences. With a lifetime risk of one in 400, MS is potentially the most common cause of disability in young adults [1]. Most patients present with the relapsing-remitting form of MS (RRMS) [2], which means that relapses with exacerbating symptoms alternate with remissions [3].

Management of RRMS requires a multimodal approach comprising both the treatment of acute relapses by corticosteroids and the suppression of disease activity by disease-modifying treatments (DMTs), including interferon beta (IFN-beta) preparations and glatiramer acetate (GA) [4]. Although early initiation and consistent administration of DMTs have been shown to decrease relapse rate and disease worsening [5-10], MS remains an incurable and debilitating disease. Since the life expectancy of MS patients is similar to that of the general population [11], MS patients require long-term therapy along with a continuous monitoring of drug efficacy, safety, and patients' satisfaction to avoid treatment-related complications and to improve treatment compliance [12,13].

Due to the wide distribution of lesions and diffuse disease processes, RRMS patients not only suffer from disability, but also from concomitant symptoms, such as visual loss, cognitive impairment, fatigue, and depression [14,15]. Quality of life declines with disease worsening [16,17]. Consequently, RRMS not only imposes severe physical hardship, but also a considerable psychosocial burden on patients, their families, and society [16]. Due to both the diversity of these physical, psychological, and social consequences and disease manifestation at a young age, RRMS is one of the most costly neurological diseases. The mean annual cost per MS patient—RRMS and progressive forms—in Europe is estimated at €27,000, which translated into total costs of €14.5 billion in 2010 [18].

This economic burden can be divided into direct and indirect costs. Direct costs represent the value of resources consumed to diagnose, treat, and accommodate MS patients with their condition, involving costs for pharmaceuticals, inpatient and outpatient care, and additional therapies such as physiotherapy. Indirect costs arise from unemployment, premature retirement, reduced productivity, and impaired quality of life [17]. In early MS stages, direct costs, predominantly for DMTs, account for the largest share of costs. Indirect costs increase during later MS stages, when disability and MS-associated symptoms become advanced [19]. There is surprisingly little comparative data on how these costs are differentially influenced by the use of DMTs in the outpatient setting. Moreover, many data sources do not include DMTs [20-23] or ignore direct nonmedical, indirect, or informal care costs [24,25]. Therefore, a detailed description of the economic burden associated with RRMS, in combination with efficacy and safety data, would be of great value for clinicians and health care providers.

Here, we present the study design of the ProspEctive phArmacoeconomic cohoRt evaluation (PEARL), a prospective, multicenter, noninterventional cohort study in RRMS patients treated with IFN-beta or GA. The 24-month observational phase of PEARL aims at collecting clinical efficacy and safety data, as well as pharmacoeconomic data on RRMS patients treated with approved DMTs in daily outpatient practice in Germany.

## Methods

### Study Design

**Figure 1:**
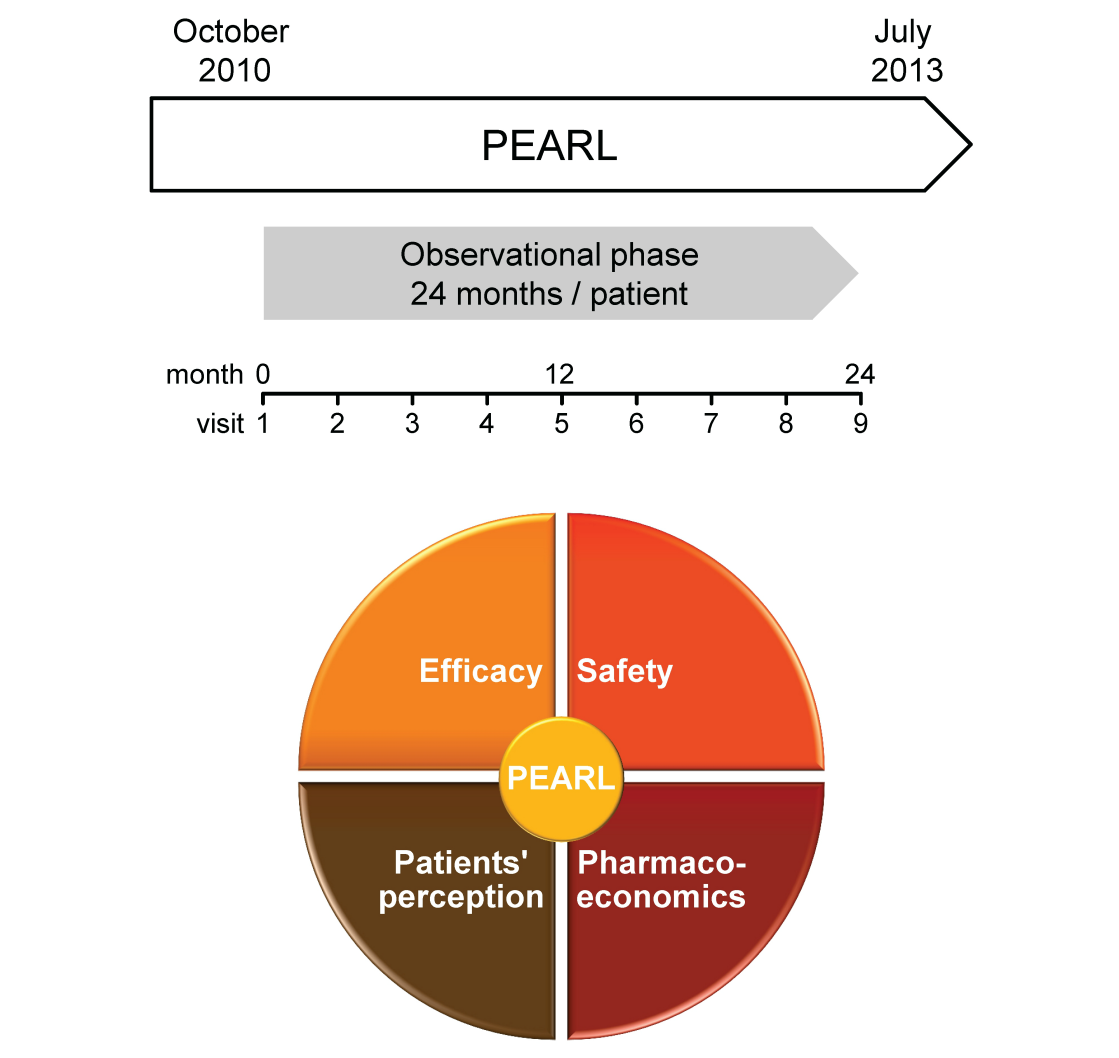
Timeline of PEARL, a noninterventional cohort study on relapsing-remitting multiple sclerosis (RRMS) patients treated with disease-modifying treatments (DMTs) in outpatient care. The study includes approximately 1800 RRMS patients from 180 neurological practices. Four major categories of data are evaluated during the observational phase of 24 months per patient (study visits 1–9). PEARL: ProspEctive phArmacoeconomic cohoRt evaluation.

PEARL is a prospective, multicenter, noninterventional cohort study of RRMS patients treated with IFN-beta or GA in daily outpatient care in Germany. The primary aim of this study was to collect and describe clinical and pharmacoeconomic data of RRMS patients treated with different DMTs. Clinical data comprised information on long-term efficacy and safety of DMTs, as assessed by clinicians and patients, and their effects on the patients' quality of life, treatment compliance, and treatment satisfaction. In this respect, data regarding the change between first-line DMTs and its impact on disease worsening were collected. Pharmacoeconomic data included information on both prescription of, and treatment with, DMTs and on resource utilization by RRMS patients. The study was designed to include 1800 patients from 180 neurological practices. The observational phase of 24 months per patient started in October 2010 and ended in July 2013 (Figure 1).

Approval from independent, local competent ethics committees was obtained, and the study was conducted in accordance with both the standards of the Association of Voluntary Self-Control of the Pharmaceutical Industry—Verein Freiwillige Selbstkontrolle für die Arzneimittelindustrie (FSA) [26]—and recommendations on the quality of noninterventional observational studies [27,28]. The study is registered as CNVF233ADE08 [29].

### Study Population

Patients with a diagnosis of RRMS were eligible if they had been continuously treated with an approved DMT—interferon (IFN) (Avonex, Betaferon, Extavia, or Rebif) or GA (Copaxone)—for at least 30 days, and if informed consent was given prior to study inclusion. There were no further selection criteria, except for the contraindications mentioned in the respective summaries of product characteristics [30-34]. The study protocol required therapeutic decisions to be made according to medical necessities, independently of study participation. To avoid selection bias, RRMS patients were enrolled in a consecutive order at each study center.

RRMS patients are normally treated in an outpatient setting. The rationale for the PEARL sample size was based on the estimated number of about 1300 neurologists in outpatient practice in Germany. Enrolling above 10% of the practices was deemed sufficient to obtain representative data. The selected number of 180 neurological practices/centers to be enrolled corresponds to 10-15% of total neurological practices and centers in Germany. Representativeness was further ensured by the regional distribution of participating practices and centers. The rationale to include 10 RRMS patients per practice was based on a practical anticipation of the practices' capabilities in study performance and their average potential to contribute patients based on expert advice. In 2010, approximately 200,000 statutory health-insured patients were diagnosed with MS—mostly RRMS and less progressive forms [35]. With an anticipated 1800 included RRMS patients, PEARL approximated 1% of German RRMS patients. With this and an observational phase of 24 months per patient, PEARL was deemed sufficient to obtain representative data on the most commonly prescribed DMTs in the outpatient setting.

### Procedures

In accordance with routine outpatient care, participants were required to attend follow-up visits every 3 months, in addition to the first visit at baseline and the final visit after 24 months. Demographic and clinical data obtained from interviews, examinations, and medical records were transferred into the case report form (CRF) by the neurologist responsible. Patient questionnaires on patient-reported resource utilization, quality of life, treatment satisfaction, and compliance were completed by participants at regular visits in the presence of a health professional. Additionally, data on adverse events (AEs) were obtained directly from the patient. All patient-reported information was documented in the CRF by the neurologist responsible.

Data quality was ensured by validation checks at the time of data entry. Data were reviewed on an ongoing basis, and queries to the study sites were automatically initiated and followed. Data management was overseen by the clinical research organization responsible (Kantar Health GmbH). In study centers with three or more patients, on-site monitoring was performed by 2 months after the inclusion of the first patient. In 10% of study centers, observational data were checked against patient records after the end of observation.

### Measures

#### Patient Characteristics

Table 1 shows the parameters to be assessed during study visits. After informed consent was given, patients formally entered the study. Demographic characteristics and patient histories, including prior and concomitant diseases and treatments, were assessed at baseline. Blood pressure and heart rate were evaluated at every visit beginning at baseline, and body weight was recorded at baseline and every 12 months.

RRMS-specific patient histories were retrospectively documented at baseline. These data included time since first symptoms, diagnosis and treatment of RRMS, the number of lesions in T2-weighted magnetic resonance imaging (MRI) as well as gadolinium-enhancing (Gd+) lesions, and the number and outcome of relapses within 12 months before study entry.

Concomitant RRMS treatments, medical and nonmedical, were documented at every visit beginning at baseline. Retrospective data on previous DMTs, including the time since start of treatment and the use of an auto-injector, were collected at baseline. Then, at every visit throughout the study, data on the current use of DMTs were documented, including the switch between DMTs and premature discontinuation of medication, as well as the reasons for discontinuation.

#### Clinical Outcome

The anamnestic number and outcome of MS relapses since the last study visit were documented at each study visit, and cerebral lesions were documented if MRI scans were available. To assess the overall clinical impression of functional impairment and disability, the Clinical Global Impression (CGI) scale [36] and the Kurtzke Expanded Disability Status Scale (EDSS) [37] were evaluated at baseline and every 3 and 6 months, respectively.

#### Adverse Events and Adverse Reactions

Occurrences of AEs, adverse drug reactions (ADRs), serious AEs (SAEs), and serious ADRs (SADRs) were evaluated by investigators at every study visit. AEs were defined as unfavorable and unintended signs or symptoms, complications, and changes of the patients’ conditions during the observational phase, irrespective of relation to treatment. SAEs comprise life-threatening or fatal events, events requiring inpatient hospitalization or prolongation of hospitalization, events leading to major incapacity, persistent or significant disability, and congenital anomaly at birth, as well as events that are otherwise medically significant. The causality of any reported ADR and SADR was categorized as *certain*, *probable*, *possible*, *not assessable*, or *missing*.

#### Patients’ Perceptions of Outcome

Patients’ perceptions of DMT effectiveness was assessed by questionnaires on disability and quality of life. Patients’ perceptions of disability were scored by means of the UK (Guy's) Neurological Disability Scale (UKNDS) [38] at baseline and every 12 months. Patients' perceptions of their quality of life were documented at baseline and every 6 months by using both the European Quality-of-Life Questionnaire (EQ-5D) [39] and the Patient-Reported Outcome Indices for MS quality-of-life (PRIMUS-QoL) and activity (PRIMUS-A) subscales [40]. Treatment satisfaction and treatment compliance were evaluated at baseline and every 3 months. Treatment satisfaction was assessed by the Treatment Satisfaction Questionnaire for Medication (TSQM-9) [41]. The compliance questionnaire focused primarily on whether, when, and how long DMT was eventually discontinued.

**Table 1:** Data obtained during PEARL^a^ study visits.

Data obtained		Data obtained at various time points (X=data were obtained)		
		Baseline (Month 0)	Follow-up	Last visit (Month 24)
**Patient characteristics**				
	Demographic data and patient history	X	N/A^b^	N/A
	Heart rate, blood pressure	X	Every 3 months	X
	Weight	X	Every 12 months	X
	MS^c^ history	X	N/A	N/A
	Concomitant nonmedical MS treatments	X	Every 3 months	X
	Prior and concomitant diseases and treatments	X	N/A	N/A
	DMT^d^ at baseline	X	N/A	N/A
	Premature discontinuation of DMT	N/A	Every 3 months	X
	Switch of DMT	X	Every 3 months	X
**Efficacy**				
	MS relapses since previous visit	N/A	Every 3 months	X
	MRI^e^ lesions	N/A	If available	N/A
	Kurtzke EDSS^f^	X	Every 6 months	X
	CGI^g^ scale	X	Every 3 months	X
Safety				
	AE^h^, ADR^i^, SAE^j^, and SADR^k^	X	Every 3 months	X
**Patients' perceptions of outcome**				
	UKNDS^l^	X	Every 12 months	X
	EQ-5D^m^	X	Every 6 months	X
	PRIMUS-A^n^ and PRIMUS-QoL^o^	X	Every 6 months	X
	TSQM-9^p^	X	Every 3 months	X
	Compliance patient questionnaire	X	Every 3 months	X
**Pharmacoeconomic data**				
	Patient resource questionnaire	X	Every 3 months	X
	Practice questionnaire	N/A	Once	N/A

^a^PEARL: ProspEctive phArmacoeconomic cohoRt evaluation.

^b^N/A: not applicable.

^c^MS: multiple sclerosis.

^d^DMT: disease-modifying treatment.

^e^MRI: magnetic resonance imaging.

^f^EDSS: Expanded Disability Status Scale.

^g^CGI: Clinical Global Impression.

^h^AE: adverse event.

^i^ADR: adverse drug reaction.

^j^SAE: serious adverse event.

^k^SADR: serious adverse drug reaction.

^l^UKNDS: UK (Guy's) Neurological Disability Scale.

^m^EQ-5D: European Quality-of-Life Questionnaire.

^n^PRIMUS-A: Patient-Reported Outcome Indices for Multiple Sclerosis activity subscale.

^o^PRIMUS-QoL: Patient-Reported Outcome Indices for Multiple Sclerosis quality-of-life subscale.

^p^TSQM-9: Treatment Satisfaction Questionnaire for Medication.

#### Pharmacoeconomic Data

Pharmacoeconomic data obtained in this study are primarily based on the analysis of the patient resource questionnaire, which was to be completed by patients at every visit beginning at baseline. In this questionnaire, several demographic data such as marital status, education, employment, and status of health and long-term care insurances were assessed by multiple-choice items. Additionally, participants were asked about their responsibility for relatives and the extent of concerns arising thereof on a 10-point Likert scale ranging from 0 (*not concerned at all*) to 10 (*maximally concerned*). The impact of RRMS on work productivity was assessed by *yes/no* questions combined with free-text fields asking about sick leaves and workplace changes. Patients were then asked to assess their productivity using another 10-point Likert scale ranging from 0 (*not affected by MS*) to 10 (*completely affected*).

*Yes/no* questions combined with free-text fields asked participants to state their financial expenditures on RRMS-specific and other medications, treatments, and devices. Patients were asked to specify outpatient therapies and medical consultations. Additional free-text fields asked for inpatient and outpatient care caused by MS relapses. Another question then asked if patients participated in nurse support programs such as EXTRACARE [42].

In addition to the patient-resource questionnaire, a practice questionnaire was completed once by each participating practice or center. This questionnaire collected information on the practice infrastructure and asked physicians and MS nurses for the number of patients treated and the time spent for diagnosis, therapy initiation, follow-up examinations, and advice. It further asked physicians to state the most important aspects mentioned in patients’ interviews. The questionnaire then assessed the physicians' perceived treatment compliance and treatment satisfaction of RRMS patients and, additionally, their presumed underlying factors.

### Statistical Analyses

All pharmacoeconomic, safety-, and effectiveness-related analyses will be performed for the full analysis set of patients, comprising patients who fulfilled all inclusion criteria mentioned above and attended at least one follow-up visit. In each analysis, the number of patients with missing data will be separately presented.

Primary statistical analyses are descriptive statistics such as mean, standard deviation, minimum, median, maximum, 25th and 75th percentiles, and number of nonmissing values. The descriptive statistical analysis will be used to summarize continuous variables, which will be additionally categorized in a clinically meaningful way. Frequency tables with absolute and relative frequencies will represent categorical data. For single efficacy parameters, such as CGI and EDSS scores as well as number of MS lesions, changes will be expressed as difference from baseline or summarized in shift tables. Subgroups defined by MS relapse will be compared using stratified analyses. For analyses, the statistical software SAS version 9.3 (SAS Institute Inc) will be used.

## Results

Data collection for PEARL started in September 2010 and ended in July 2013. As of July 2015, the study is completed and is currently being analyzed and written up.

## Discussion

### Principal Findings

In this paper, we describe the study protocol of PEARL, a prospective, multicenter, noninterventional cohort study on RRMS patients treated with DMTs in daily outpatient practice in Germany. The aim of this study was to collect clinical and pharmacoeconomic data of a representative cohort of 1800 RRMS patients treated with different DMTs under routine outpatient conditions. The data therefore includes efficacy and safety-related outcome parameters as assessed by clinicians and patients, as well as pharmacoeconomic data of RRMS patients, including information on employment, work productivity, and resource utilization.

When assessing efficacy and safety, PEARL placed emphasis on the patients’ perceptions. The patients’ perceptions of their disability and health-related quality of life might differ from those of the attending physicians [43,44]. Therefore, evaluating the patients’ perspectives is meaningful in improving the quality of treatment and health care services [12]. We further collected data on the frequency and reasons why patients switch DMTs and, moreover, assessed treatment satisfaction of RRMS patients. This information is clinically relevant because higher treatment satisfaction correlates with higher treatment compliance [13]; in turn, better compliance correlates with improved outcomes. It has been shown that the number of relapses can be reduced, and functional and cognitive abilities as well as quality of life can be improved, when RRMS patients comply with treatment in the long term [45,46]. Adherence to treatment has been demonstrated to reduce the risk of MS relapses and disability [47]. Since adherence rates in clinical trials seem to be higher than in routine care, the detailed assessment of treatment satisfaction and compliance in a real-world setting is necessary [48].

RRMS is obviously associated with significant economic burdens. Direct and indirect costs increase during relapses and disease worsening [49]. If new therapies are able to reduce relapses, delay accumulation of disability, relieve symptoms, and allow an acceptable quality of life, the overall economic burden on individuals and society might be reduced. Furthermore, the savings in indirect costs might outweigh direct costs of treatment. However, the current state of research in this area is subject to several uncertainties and data gaps. For example, most studies on the economic aspects of MS had been conducted before DMTs were established as standard treatment, and results may no longer apply [20-23]. Moreover, results of other cost studies might not be representative because of the selection criteria applied. Our study therefore evaluated pharmacoeconomic information in combination with efficacy/safety data of different DMTs currently used in routine outpatient care. Except for contraindications to DMTs and the requirement that informed consent be obtained, no selection criteria were applied in our study to achieve the best possible representativeness.

### Conclusions

The results of the PEARL study will add useful information to the currently incomplete data on the health status and resource utilization of RRMS patients treated with IFN-beta or GA in routine outpatient care in Germany. From both the economic and the clinical points of view, the results of PEARL will provide further insight into the health care costs and benefits of RRMS therapy and might support health care providers who are seeking ways to offer more cost-effective care.

## Abbreviations

ADR: adverse drug reaction
AE: adverse event
CGI: Clinical Global Impression
CRF: case report form
DMT: disease-modifying treatment
EDSS: Expanded Disability Status Scale
EQ-5D: European Quality-of-Life Questionnaire
FSA: Freiwillige Selbstkontrolle für die Arzneimittelindustrie
GA: glatiramer acetate
Gd+: gadolinium enhancing
IFN: interferon
IFN-beta: interferon beta
MRI: magnetic resonance imaging
MS: multiple sclerosis
N/A: not applicable
PEARL: ProspEctive phArmacoeconomic cohoRt evaluation
PRIMUS-A: Patient-Reported Outcome Indices for Multiple Sclerosis activity subscale
PRIMUS-QoL: Patient-Reported Outcome Indices for Multiple Sclerosis quality-of-life subscale
RRMS: relapsing-remitting MS
SADR: serious adverse drug reaction
SAE: serious adverse event
TSQM-9: Treatment Satisfaction Questionnaire for Medication
UKNDS: UK (Guy's) Neurological Disability Scale
